# RNA-seq-based genome annotation and identification of long-noncoding RNAs in the grapevine cultivar ‘Riesling’

**DOI:** 10.1186/s12864-017-4346-6

**Published:** 2017-12-02

**Authors:** Zachary N. Harris, Laszlo G. Kovacs, Jason P. Londo

**Affiliations:** 1 Missouri State University, Biology Department, 901 S. National Ave, Springfield, MO USA; 2 United States Department of Agriculture, Agricultural Research Service, Grape Genetics Research Unit, 630 W. North Street, Geneva, NY USA; 3 Present address: Saint Louis University, Department of Biology, 1 N. Grand Blvd, Saint Louis, MO USA

**Keywords:** Transcriptome, Genome re-annotation, RNA-seq, lncRNA, Minimum free energy, Riesling, *Vitis vinifera*

## Abstract

**Background:**

The technological advances of RNA-seq and de novo transcriptome assembly have enabled genome annotation and transcriptome profiling in highly heterozygous species such as grapevine (*Vitis vinifera* L.). This work is an attempt to utilize a de novo-assembled transcriptome of the *V. vinifera* cultivar ‘Riesling’ to improve annotation of the grapevine reference genome sequence.

**Results:**

Here we show that the transcriptome assembly of a single *V. vinifera* cultivar is insufficient for a complete genome annotation of the grapevine reference genome constructed from *V. vinifera* PN40024. Further, we provide evidence that the gene models we identified cannot be completely anchored to the previously published *V. vinifera* PN40024 gene models. In addition to these findings, we present a computational pipeline for the de novo identification of lncRNAs. Our results demonstrate that, in grapevine, lncRNAs are significantly different from protein coding transcripts in such metrics as length, GC-content, minimum free energy, and length-corrected minimum free energy.

**Conclusions:**

In grapevine, high-level heterozygosity necessitates that transcriptome characterization be based on cultivar-specific reference genome sequences. Our results strengthen the hypothesis that lncRNAs have thermodynamically different properties than protein-coding RNAs. The analyses of both coding and non-coding RNAs will be instrumental in uncovering inter-cultivar variation in wild and cultivated grapevine species.

**Electronic supplementary material:**

The online version of this article (10.1186/s12864-017-4346-6) contains supplementary material, which is available to authorized users.

## Background

RNA sequencing (RNA-seq) has emerged as a powerful technology for an in-depth, genome-wide view of the transcriptome. In grapevine, *Vitis vinifera* L., RNA-seq-based transcriptome profiling has been used to interrogate the molecular underpinning of such diverse biological phenomena as photosynthesis [1], berry development [2–4], tissue maturation [5], plant-pathogen interactions [6–8] and environmental effects [9]. A thorough understanding of grape biology is important for the development of new cultivars and the fine-tuning of cultural practices to meet the challenges of the changing climate. Furthermore, grapevine is becoming the model species for woody perennial plants, therefore grape genomic information can be leveraged well beyond its application in viticulture.

To transform RNA-seq data into biologically meaningful information, raw sequence reads are assembled into transcripts. These transcripts are, in turn, anchored to transcripts of the same or a different organism. This process is facilitated by a well-annotated reference genome sequence that can be used in varieties and accessions across a species. In grapevine, the comprehensive annotation of the reference genome sequence has proven difficult. Recent reports have demonstrated that a large portion of gene-models in the reference genome sequence cannot be anchored to newly assembled genomic or transcriptomic data. For example, the recently sequenced ‘Cabernet Sauvignon’ genome identified only 57% of the gene models in the 12Xv1 ‘Pinot Noir’-derived PN40024 reference genome annotation [10], and transcriptome analyses in ‘Corvina’ identified only 51% of the 12Xv1 gene models [2]. A likely reason for this is the high-level heterozygosity of grapevine which encompasses a broad genetic diversity even among its most commonly grown varieties [11]. Thus, most RNA-seq reads from a given grape variety are allelic variants of the reference genome sequence, which represents a single haplotype of a single ‘Pinot Noir’-derived genotype. Similarly, most RNA-seq reads are allelic variants of annotated transcripts when the annotation is based on the transcriptome of a different variety. In instances where the extent of allelic divergence is substantial, there is an increased probability that RNA-seq reads are assembled into false chimeric transcripts or an assembly can be erroneously identified as a novel paralog in the genome. Alternatively, allelic variations may be interpreted as sequencing errors, particularly in low-level expressed transcripts. The increased probability of such artifacts can lead to the construction of an incorrect transcriptome.

RNA-seq is not limited by previously identified genetic information, but has the capacity to detect all transcribed sequences, including non-protein-coding transcripts. This lead to the discovery, in both plants and animals, of an entire new class of long non-coding RNA species (lncRNAs) in addition to the known ribosomal, transfer, short nuclear and short cytoplasmic RNA species. The new class of lncRNAs are operationally defined as 200-nt or longer transcripts that can be capped, spliced, and poly-adenylated, but that do not typically contain an open reading frame. Plants express thousands of lncRNAs, but only a handful of them have been experimentally validated [12–16]. The few validated lncRNAs were found to play a role in the regulation of such processes as vernalization, photomorphogenesis, phosphate homeostasis, and auxin-mediated gene expression regulation in *Arabidopsis thaliana* (reviewed by [17, 18]). Even fewer lncRNAs have been associated with a regulatory role in other plant species [18], and nothing is known about the function of lncRNAs in grapevine.

Here, we present a reannotation of the PN40024 reference genome sequence based on the transcriptome of a single *Vitis vinifera* cultivar. In an attempt to mitigate the problems associated with high-level heterozygosity, and to increase the probability of identifying novel transcripts, we constructed this annotation by taking a de novo transcriptome assembly approach. To catalog as many of the grape transcripts as possible, we used ‘Riesling’ RNA-seq libraries that collectively represented a broad range of grapevine tissues. Moreover, we present a pipeline for the de novo identification of long non-coding RNA entirely independent of a reference genome. This pipeline is then applied to *V. vinifera* cv. ‘Riesling’ for the first characterization of lncRNAs in the cultivar.

## Results

### Genome annotation

The *Vitis vinifera* cv. ‘Riesling’ transcriptome was assembled from RNA-seq reads derived from two accessions of this cultivar, 588,673 and Ventosa. Quality filtering and trimming resulted in 14,190,809 paired end reads for 588,673. Following quality control, reads were re-paired using the program pairfq_lite [19]. Pairfq_lite returned 6,679,255 reads with a paired read on both strands, 514,591 reads unpaired on the forward strand, and 317,708 reads unpaired on the reverse strand. Of the paired reads, 91.45% aligned to the *Vitis vinifera* PN40024 12Xv2 reference genome sequence hosted at Unité de Recherche Génomique Info (URGI) [20]. Of the unpaired reads, 79.45% aligned to the reference genome, leading to a total alignment rate of 79.74%. Both paired and unpaired reads were assembled using the de novo transcript assembler Trinity (v2.0.6) [21, 22]. Trinity assembled 62,745 contigs with an average contig length of 859 nt and a median contig length of 551 nt. The contig N50 for the assembly was 1325 nt. The 62,745 contigs assembled were represented by 49,330 clusters (putatively labeled as “genes” by Trinity). Quality control and trimming resulted in 103,677,027 reads for the Ventosa accession. Pairfq_lite [19] returned 48,639,916 reads paired on both strands, 4,393,048 reads unpaired on the forward strand, and 2,004,147 reads unpaired on the reverse strand. Of the paired and unpaired reads, 91.14% and 77.06% aligned, respectively, to the reference genome, resulting in a total alignment rate of 80.64%. Trinity identified 157,779 contigs with an average length of 840 nt and median length of 373 nt. These 157,779 contigs were clustered into 109,215 Trinity-identified clusters. The N50 for the assembly was 1434 nt. Additional statistics of the Trinity assemblies are presented in Table 1.

**Table 1:** Additional metrics describing the Trinity assemblies of the accessions Ventosa and 588,673

Metric	‘Riesling’ Accession	
	Ventosa	588,673
N10	3337	2953
N20	2599	2301
N30	2130	1912
N40	1762	1603
N50	1434	1325
E10N50	1413	1008
E20N50	1349	1119
E30N50	1290	1149
E40N50	1413	1259
E50N50	1520	1345
E60N50	1671	1480
E70N50	1815	1612
E80N50	1947	1620
E90N50	1922	1499
E100N50	1437	1328

All transcripts from both accessions were used for a complete genome annotation of the *V. vinifera* PN40024 12Xv2 reference genome sequence, using the program Maker [23, 24]. Using these transcripts and the entire Uniprot-Swiss-Prot reference protein database, 65,342 putative gene models were identified. Gene models first identified by Maker were then assigned to proteins in the Uniprot-Swiss-Prot reference database [25] using the blastp algorithm of the BLAST v.2.2.29 software suite [26]. This operation linked 1680 gene models to proteins, 1004 of which were carried forward from the Uniprot database itself in the annotation.

**Fig. 1:**
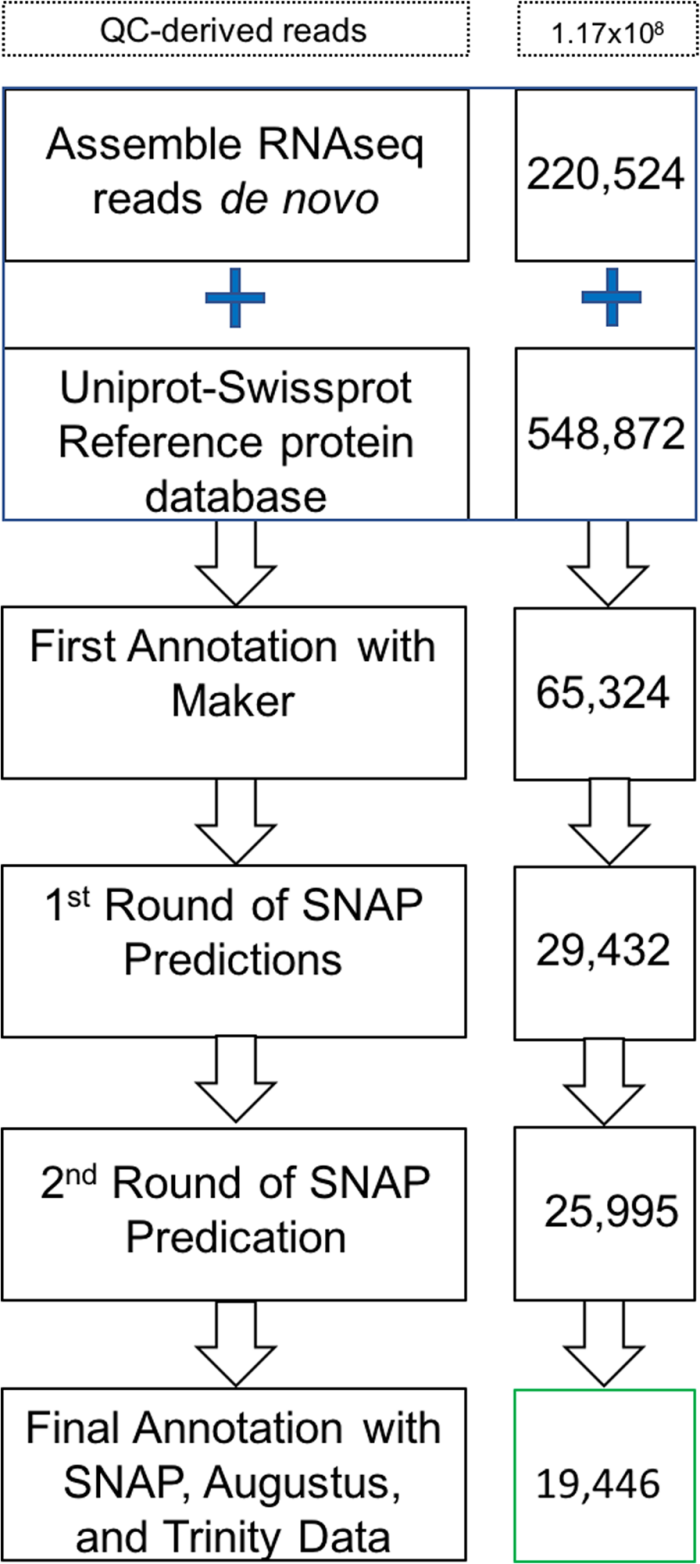
Diagrammatic representation of the steps taken to annotate the 12Xv2 PN40024 reference genome. The numbers on the right indicate the number of transcripts (or proteins in the case of Uniprot-Swiss-Prot) that were fed into or derived from the step to the left. The final number, framed in green, shows the number of gene models in the final annotation

Using the combined accession output from Maker, gene models were used to train the SNAP gene prediction algorithm [27]. Raw output was used to train the first pass of SNAP, and this output was used to train the second pass. This resulted in a statistical model, Riesling.hmm [see Additional file 1]. This model was then coupled to the statistical model derived from the gene predictor algorithm Augustus [28] trained with *Arabidopsis thaliana* data. These models were then re-introduced to the genome sequence, this time in the context of the raw Trinity transcripts. The combination of Maker, SNAP, and Augustus predicted the presence of 19,446 gene models that were supported by RNA-seq evidence [see Availability of Data and Materials]. The sequence of steps for the annotation of the genome is depicted in Fig. 1. These annotated gene-models had a Benchmarking Universal Single-Copy Ortholog (BUSCO) score of 59.4%, indicating a ~60% recovery of the predicted *V. vinifera* transcriptome.

### Functional annotation of the gene models

**Fig. 2:**
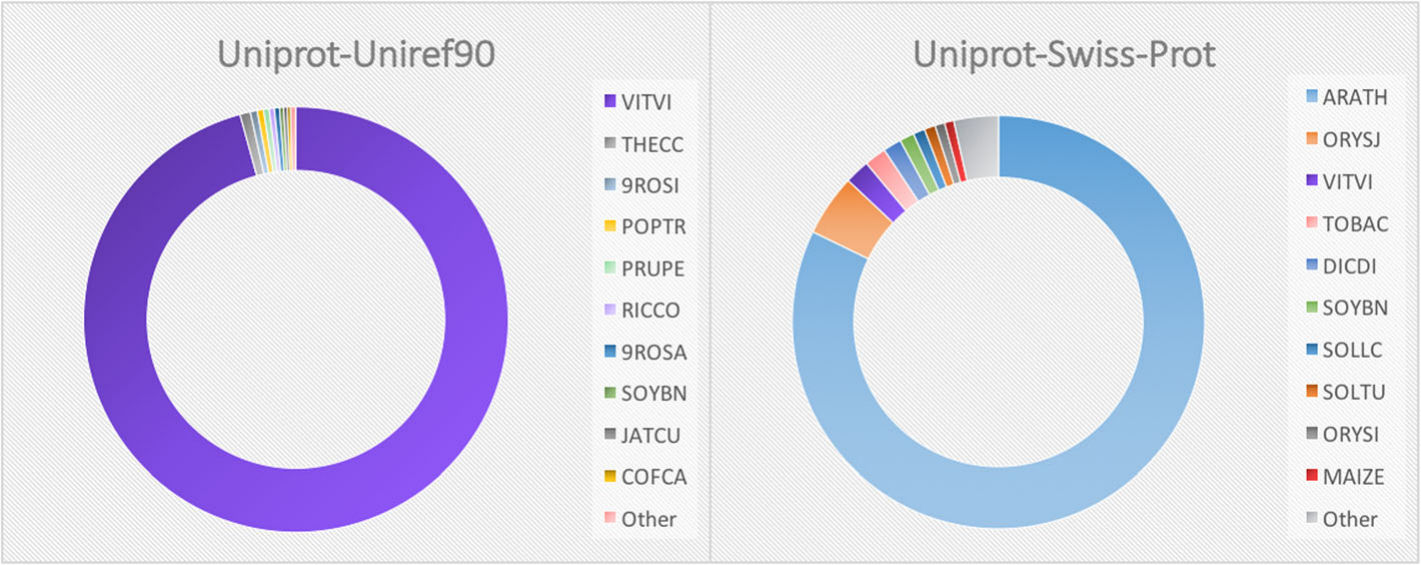
Most common BLAST targets in protein comparisons of the gene-model annotation to two reference protein databases (Uniprot-Swiss-Prot and Uniprot-Uniref90) at E-value threshold of 1 × 10^−20^. The species codes are Uniprot species IDs (http://www.uniprot.org/docs/speclist). Color selection is random with the excpetion of VITVI and SOYBN which are repesented by purple and green, respectively, in both figures

Predicted protein domains were searched against the reference protein domain database Pfam31.0 [29] using the program hmmscan in the HMMER v3.1b2 software suite [30]. Hits were considered significant if they matched with an expected value (E-value) of less than or equal to 1 × 10^−05^. Under this threshold, 26,287 protein domains were identified. This cohort was composed of 3721 unique domains identified across 13,942 unique gene models. In many cases, Pfam domains could be tied to Gene Ontology (GO) [31] terms, classes, and functions using a custom boilerplate SQLite database. In total, 1742 Pfam domains could be tied to 3713 unique GO terms for a total of 27,823 tied instances. In total, 16,598 (59.7%), 8645 (31.1%), and 2582 (9.3%) instances of the molecular function, the biological process, and the cellular component classes were tied, respectively [see Additional file 2]. Proteins were further functionally annotated using blastp against the Uniprot-Swiss-Prot and Uniprot-Uniref90 [32] databases. Annotation against Uniprot-Swiss-Prot anchored 14,866 and 12,572 proteins with E-values of less than or equal to 1 × 10^−05^ and 1 × 10^−20^, respectively. Annotation against Uniprot-Uniref90 anchored 18,535 and 17,970 proteins with an E-value of 1 × 10^−05^ and 1 × 10^−20^, respectively [see Additional file 3]. The most frequent species from which the BLAST homologies were identified are listed in Fig. 2.

### Anchoring gene models to the legacy transcriptomes

In order to anchor newly annotated transcripts to the legacy *V. vinifera* PN40024 v2.1 transcriptome [33] (filtered to only use the top isoform for each transcript), we devised an iterative approach for reciprocal best hit (iRBH) analysis based on the assumption that each gene is present in the same number of copies in both ‘Riesling’ and PN40024. Though this working assumption may not be correct for all gene families, it is likely to be correct for the majority of genes in light of the pronounced karyotypic conservation and interfertility across *V. vinifera* accessions [34]. This analysis sorted the forward and backward blastp results in such a way that each transcript only matched each unique target one time. The following processes then occurred iteratively: (1) BLAST results were sorted such that only the highest scoring hit for each query was kept, (2) RBHs were identified, and 3) a gene model labeled as a RBH (either target or query) was removed from further analysis at every incidence of the BLAST output.

This process was executed iteratively 25 times, whereby 16,600 gene models were putatively anchored to the legacy transcriptome with a threshold E-value of 1 × 10^−05^. Of the models anchored to the v2.1 transcriptome, 15,364 were identified on the same chromosome in both annotations. Over half of the models that differed in chromosomal location (677) were assigned to chrUkn (a compilation of scaffolds that cannot be assigned to any of the 19 grape chromosomes) in either the legacy or the present annotation. Those models that differed in chromosomal location, but were not assigned to chrUkn in either annotation, had overall a lower bit-score/length (v2.1 model) ratios (0.82 vs. 1.64) and marginally higher E-values (7.67 × 10^−23^ vs. 6.75 × 10^−24^) than those that found anchors on the same chromosome [see Additional file 3].

Gene-models from the v2.1 annotation that returned no “reciprocal good hits” were labeled as unsupported models. Gene models that were annotated by either Maker, SNAP, or Augustus, but that lacked RNA-seq support, were scanned for these models using a single RBH and chromosomal location. In total, 15,245 v2.1 gene models lacked Maker anchors. Of the 9558 models not supported by RNA-seq, 8886 models found RBHs at an E-value threshold of 1 × 10^−05^, and 8047 RBHs occurred on the same chromosome. Of those 1505 models that differed in chromosomal location, 559 models were assigned to chrUkn in either the v2.1 or Maker annotation.

### Gene duplication

Using MCScanX [35], duplicated genes were identified using a self-BLAST-based collinear approach at various E-values. Gene duplication was initially classified using the MCScanX tool duplicate_gene_classifier, whereby 17,115 genes were considered the results of whole genome duplication. Furthermore, 1360, 226, and 715 genes were considered dispersed, proximal, and tandem duplicates, respectively, and 30 genes were considered singletons [see Additional file 3]. Because genes can represent more than one of these gene types, all evidence of duplication was explicitly searched in the gene models at various E-value thresholds. At a threshold E-value of 1 × 10^−20^, evidence was found for 20,563 gene duplication pairs across 11,925 genes. MCScanX was also used to detect all tandemly arrayed genes (TAGs). Regardless of threshold E-value, 2281 tandem duplications were identified across 3480 (17.9%) unique genes. Of these, 1928 (55%) appeared in arrays of at least 3 genes, and 1552 in 2-gene arrays.

### Design of a de novo pipeline for lncRNA identification

**Fig. 3:**
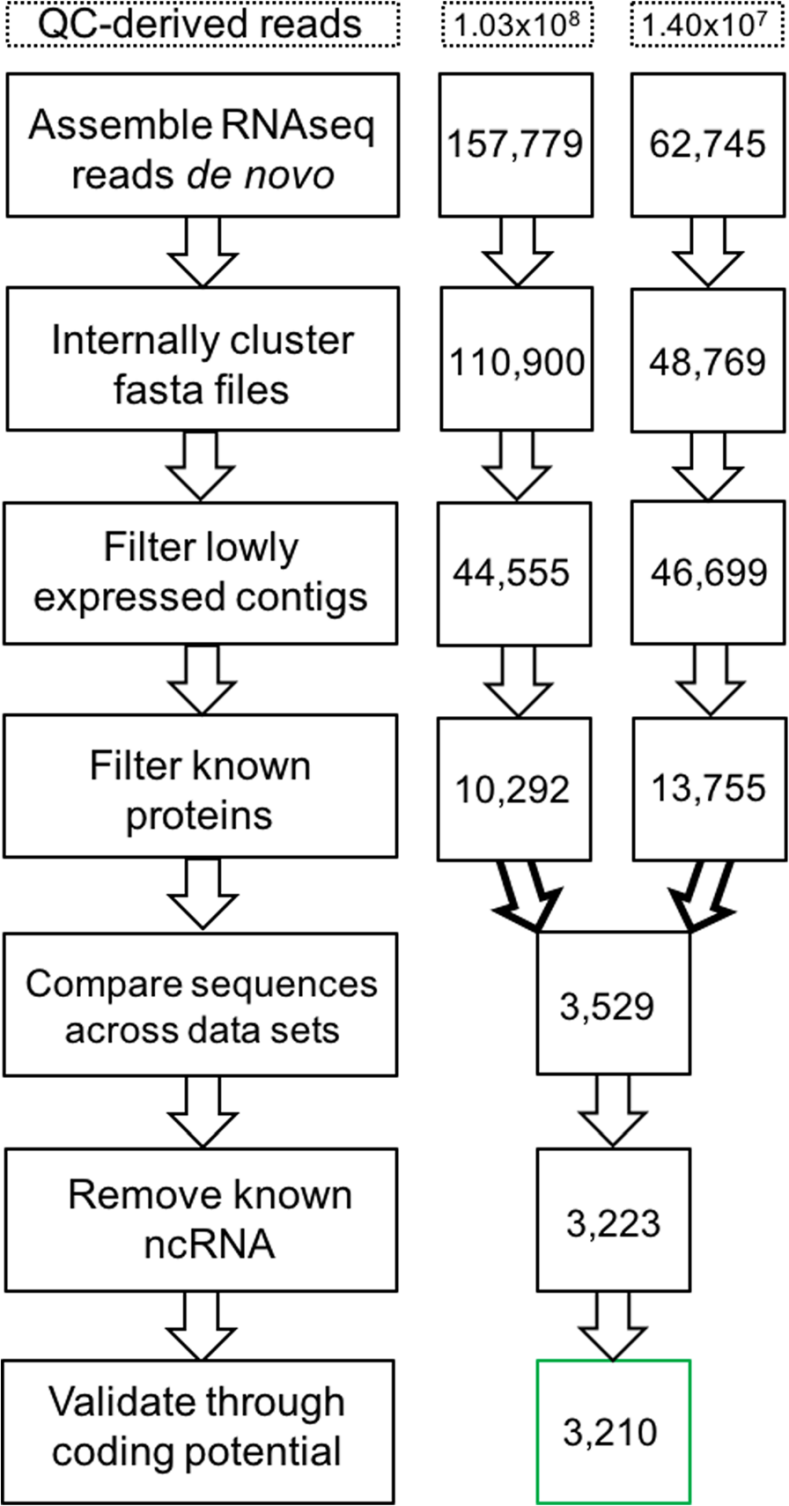
Diagram of the long non-coding RNA identification pipeline. The numbers on the left of the figure indicate the number of transcripts remaining after the filtering step shown on the right. Through the ‘Compare sequences across data sets’ step, numbers are shown for accessions Ventosa and 588,673 respecitvely. The final number, framed in green, shows the number of psuedo-validated lncRNAs through the Coding Potential Calculator

A computational pipeline was constructed to glean lncRNAs from assembled transcriptomes. To make the pipeline broadly applicable, it was designed to identify lncRNAs from raw RNA-seq reads in a reference genome-independent manner. The essential function of the pipeline was to remove protein-coding transcripts and short non-coding RNA sequences. First, Trinity [21, 22] was used to assemble raw RNA-seq reads into a set of transcripts, which was then purged of redundantly identified transcripts using CD-HIT [36]. Clustered transcripts were then filtered by expression level via RSEM [37], and the remaining set was further filtered to remove known protein coding genes identified by BLAST using Trinotate v2.0.2 [38]. Finally, various sets of non-protein-coding transcripts extracted from independent RNA-seq data of the same grape genotype were juxtaposed retaining only transcripts present in multiple sets to ensure a low false positive identification rate [39]. These final transcripts were then searched against the reference RNA database Rfam [40] using Infernal [41] to remove known ncRNAs and validated using the Coding Potential Calculator (CPC) [42]. Fig. 3 is a diagrammatic representation of this pipeline.

### lncRNA identification pipeline

Due to the highly redundant nature of de novo assembled transcriptome builds, the Trinity output was clustered for both ‘Riesling’ accessions 588,673 and Ventosa using the cd-hit-est. algorithm implemented by the CD-HIT software suite. Clustering in accessions 588,673 and Ventosa resulted in 48,769 and 110,900 contigs, respectively. To further reduce the complexity of the data sets, clustered transcript sets were filtered by an expression level threshold of FPKM > = 1.50. Accessions 588,673 and Ventosa resulted in 46,699 and 31,103 contigs, respectively.

To remove all transcripts putatively annotated as protein-coding from the clustered and expression-level filtered transcript sets, we employed the Trinotate pipeline. In preparation, all transcripts in both data sets were translated to proteins using the tool TransDecoder [43]. RNA sequences were then searched using the blastx algorithm and translated proteins were searched using the blastp algorithm against the UniProt-Swiss-Prot and Uniprot-Uniref90 reference protein databases using an E-value threshold of 1 × 10^−20^. Only the top BLAST hit for each sequence in the accessions was accepted based on bit score, E-value, and percent identity. Contigs were binned into categories of Viridiplantae proteins, non-plant proteins, and proteins for which no homologous hit was found. For 588,673, the number of transcripts identified to encode Viridiplantae proteins, non-plant proteins, and proteins with no homology were 41,686, 3340, and 13,755, respectively. For Ventosa, 34,321 transcripts were identified to encode Viridiplantae proteins, 682 to encode non-plant proteins, and 10,292 to encode no homologous protein in the database.

To identify RNA sequences that occurred in both accessions, blastn was used with default parameters. Only the top BLAST hit for each contig in the Ventosa accession was accepted based on bit score, E-value, and percent identity. Only matches that had an alignment length of at least 200 nt were carried forward in the analysis. In matches of at least 200 nt, the longest transcript from either 588,673 or Ventosa was taken. This resulted in 3529 sequences.

These 3529 putatively identified non-coding RNAs then were filtered for the presence of known non-coding RNAs housed in the RFAM [40] v12.0 database via the cmscan tool in the Infernal suite v1.1.1 [41]. Using cmscan, 196 sequences were considered significant based on the E-value threshold of 0.01 and were removed from the data set. This resulted in 3223 putatively labeled long non-coding RNAs [see Availability of Data and Materials].

In order to validate the putatively labeled long non-coding RNAs, we used the program Coding Potential Calculator (CPC) [42]. Using this tool against the UniProt-Swiss-Prot database, 3210 sequences were predicted to be non-coding, substantiating the predictions generated by our pipeline. Alignment of the predicted lncRNAs to the reference genome sequence lead to 3049 mapped transcripts.

### Comparison of lncRNAs and protein coding RNAs

**Fig. 4:**
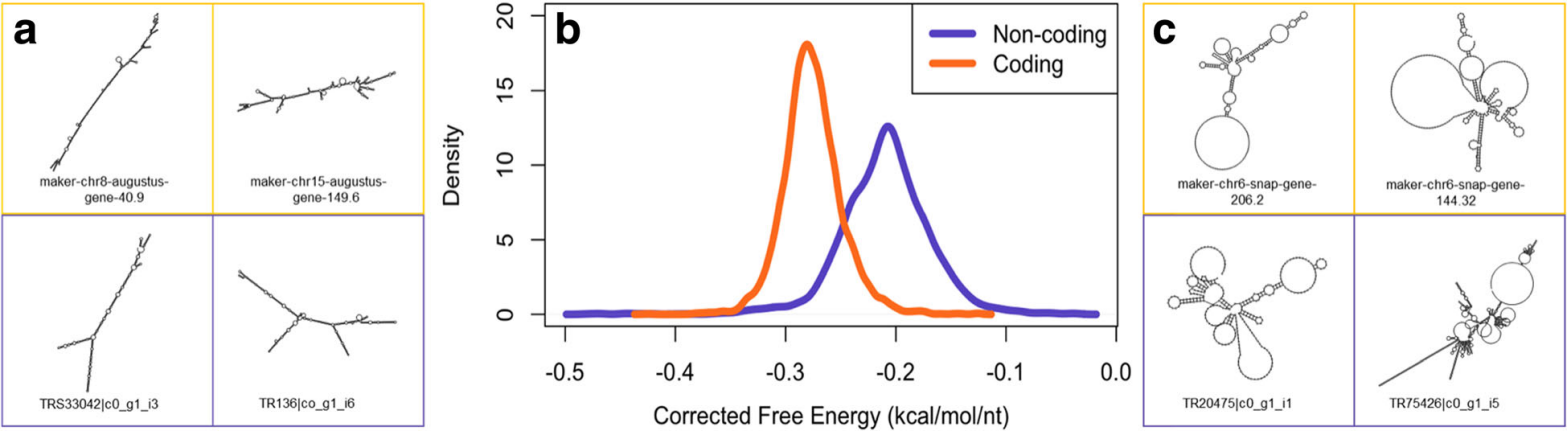
Minimum free energy structures and free energy distributions for non-coding and coding transcripts. **a** Characteristically stable (low free energy) minimum free energy structures for predicted coding (orange) and non-coding (purple) transcripts. **b** Free energy distributions for long non-coding and coding transcripts (*p* < 2.2 × 10^−16^; d = 2.05). **c** Characteristically unstable (high free energy) structures for predicted coding and non-coding transcripts

**Fig. 5:**
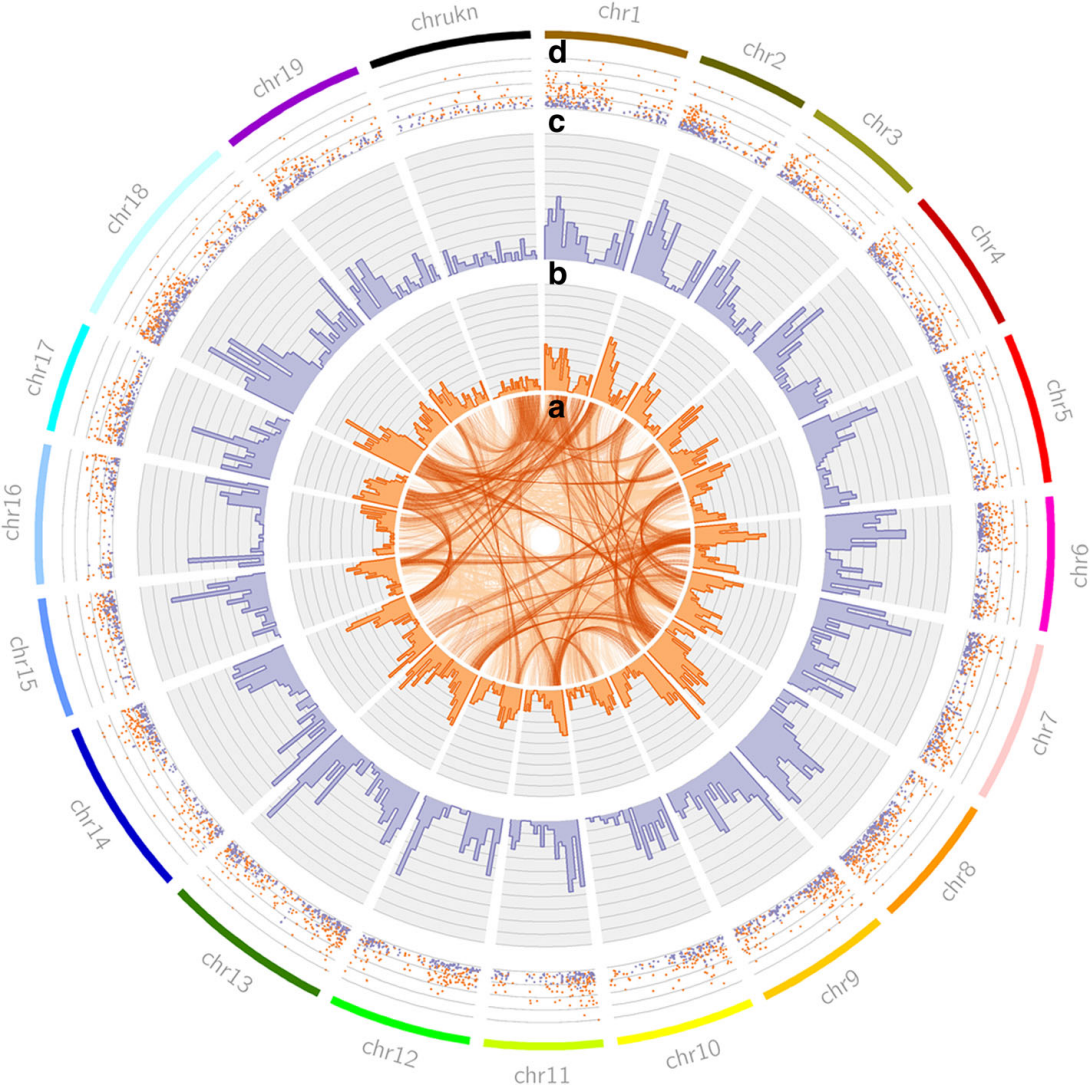
Duplications, gene density, and free energy for genes anchored to genomic locations. **a** Evidence of gene duplications in the gene annotation at two E-value thresholds: 1 × 10^−20^ and 1 × 10^−50^ (light and dark orange, respectively). **b** Frequency distributions of 3049 randomly selected protein coding genes using a weighted sampling schema binned into 1 Mbp bins. **c** Long non-coding RNA frequency of all lncRNAs that aligned to the reference genome binned into 1Mbp bins. **d** Raw (uncorrected) free energy values for 3049 lncRNAs that aligned to the reference genome (purple) and 3049 randomly selected protein coding genes (orange) (*p* < 2.2 × 10^−16^)

It has been previously observed by others that the secondary structure of lncRNAs tend to have higher free energy (less stable conformation) than protein-coding mRNAs [12, 44, 45]. To examine if grape lncRNAs identified in this study have a higher free-energy level than mRNAs, we used the RNA free energy calculator and folding algorithm RNAfold of the ViennaRNA-2.2.5 software package [46]. RNAfold was used to predict the secondary structure and the minimum free energy of all putative lncRNAs that aligned to the reference genome and a randomly selected set of 3049 annotated protein-coding transcripts identified by Maker. Secondary structures of sequences representing the highest and the lowest free energies are shown in Fig. 4. Free energy values of secondary structures were corrected for the length of the sequence. The corrected minimum free energy distribution of all analyzed RNAs are shown in Fig. 4. The mean length-corrected minimum free energy for annotated protein coding genes was −0.276 kcal/mol/nt with a standard deviation of 0.026 kcal/mol/nt. The mean length-corrected minimum free energy content of the putatively annotated lncRNAs was −0.210 kcal/mol/nt with a standard deviation of 0.041 kcal/mol/nt. The means of these two groups were found to be significantly different using a two-tailed Welch’s t-test whereby *p* < 2.2 × 10^−16^. To assess whether or not this difference was substantive, the effect size (Cohen’s *d*) was derived from the *t*-statistic. The resulting *d* value of 2.08 indicated a ‘large’ effect size of the difference [47]. The result of a Kolmogorov-Smirnov test demonstrated that the two groups were sampled from different distributions at *p* < 2.2 × 10^−16^. Additionally, both length of the transcripts and the GC-content were found to be significantly different (*t*-test, *p* < 2.2 × 10^−16^) and to be sampled from different populations (KS-test, *p* < 2.2 × 10^−16^). Thus, our data confirm previous reports that lncRNAs fold into secondary structures of lower free energy than protein-coding mRNAs do. Genomic distribution of lncRNAs and raw free energy computations are shown in Fig. 5.

## Discussion

The purpose of this work was to create a novel annotation of the 12Xv2 *V. vinifera* PN40024 reference genome sequence using expression support exclusively from *V. vinifera* cv. ‘Riesling’. To mitigate the problems associated with the high-level heterozygosity of grapevine, we have taken a de novo approach to assembling the ‘Riesling’ transcriptome, and coupled it with sequence information from the 12Xv2 reference genome sequence to generate a novel annotation for *V. vinifera*. Furthermore, to detect as many transcripts as possible, we used ‘Riesling’ RNA-seq libraries that collectively represented a broad range of grapevine tissues, including root, dormant bud, leaf, tendril, rachis, flower, and unripe and post-veraison berry. We identified 19,446 gene models that had various levels of RNA-seq support. In attempted functional annotation, we found that 13,942 (71.7%) of these gene models contain at least one Pfam domain and that 14,886 (76.4%) models have significant homology to a protein in the Uniprot-Swiss-prot database. These proportions are similar to those reported for the v2.1 reference transcriptome indicating that the function of a large segment of grapevine transcriptome cannot be predicted based on currently available data. This is not a grapevine-specific problem: results of recent plant transcriptome annotation efforts indicated that our knowledge of gene function in higher plants is still limited. For example, a recent transcriptome analysis in rose-scented geranium (*Pelargonium graveolens*) and giant cane (*Arundo donax*) found protein homology to only 66% and 55% of the transcripts [48, 49], respectively. Collectively, plant de novo transcriptome analysis studies confirmed that, despite the wealth of genomics and bioinformatics resources accumulated, our understanding of plant biology is still hindered by our inability to assign even tentative function to a large number of plant genes.

Validation of the transcriptome, using BUSCO, identified 59.4% of the expected 1440 single-copy embryophyta orthologs. This result suggested that we have recovered about 60% of the ‘Riesling’ transcriptome. This is in agreement with previous estimates that the grapevine genome contained about 30,000 genes. Even though only a subset of the v2.1 gene models were identified, several quantitative features of the ‘Riesling’ transcriptome were similar to those of other plants. For example, of the genes predicted, 17.9% (3480 genes) occurred in tandemly duplicated gene arrays (TAGs), a percentage consistent with *Arabidopsis thaliana* (16.6%) [50] and even such distantly related organisms as human, mouse, and rat (14–17%) [51].

The fraction of the transcriptome we captured is similar in range to that reported by Venturini et al. [2] who characterized a transcriptome for *V. vinifera* cv. ‘Corvina’, and was able to recover only 51% of the v1 reference annotation’s 29,971 gene-models. These results raised the question of why such a low percentage of RNA-seq-supported *V. vinifera* transcripts could be anchored to the gene models of the reference transcriptome of the same species. Venturini et al. [2] speculated that this might be due to different assortment of genes expressed in the reference and the de novo transcriptomes. While differential expression certainly plays a part, we contend that the high-level genetic diversity within *V. vinifera* is an even greater source of the difficulty in anchoring transcripts among varieties. Strong support for the this contention was provided by a recent genome assembly by Chin and co-workers [10] who assembled the genome of *Vitis vinifera* cv. ‘Cabernet Sauvignon’. Chin et al. were able to align only 16,981 (57%) of the v1 reference transcriptome’s 29,971 gene-models to the ‘Cabernet Sauvignon’ genome. As they have worked with genomic sequence alignments only, their anchoring could not have been influenced by differential gene expression. Nonetheless, their anchoring success rate of 57% was similar to that (51%) obtained by Ventirini et al. [2], who worked with de novo assembled transcripts. Importantly, both Venturini et al. [2] and Chin et al. [10] achieved these results by attempting to identify corresponding genes between varieties of the same species, *V. vinifera*.

The high level of heterozygosity within cultivated *V. vinifera* varieties is well documented and is attributed primarily to the large effective population size of the ancestral *Vitis vinifera ssp. sylvestris*, and the resultant extensive chromosomal recombination Using a grapevine genotyping array, Myles et al. [52] showed that the *V. vinifera* genome was composed of short haplotype blocks which reflect chromosomal recombination over a long evolutionary time [52]. Myles et al. also demonstrated that nearly a quarter of the polymorphisms for which *V. vinifera* varieties segregate are shared with the North American *Vitis* species. This suggested vast effective population sizes which lead to the maintenance of genetic diversity from deep ancestry. We propose that this genomic diversity within *V. vinifera* manifests itself in such high-level allelic diversity that the accurate anchoring of many gene models is not possible across varieties.

This problem could have been exacerbated by the inclusion into the v2.1 annotation of transcript information from two rootstock cultivars, the genomes of which had been derived to a great extent from non-vinifera grape genotypes. These rootstocks were collectively bred from three North American *Vitis* species, namely *Vitis berlandieri, Vitis rupestris,* and *Vitis riparia,* in addition to *V. vinifera.* The divergence time between the three North American and the Eurasian vinifera clades was estimated to be 11.12 (16.58–6.59) million years, whereas the divergence within the ancestral *V. vinifera* ssp. *sylverstris*, the wild progenitor of *V. vinifera*, occurred during a considerably shorter evolutionary time [53]. Thus, the inclusion of *V. berlandieri, V. rupestris,* and *V. riparia* transcripts in the v2.1 annotation likely introduced genetic divergence well beyond the already great divergence in *V. vinifera* itself.

Results of the transcriptome analysis presented here, supported by previous transcriptome and genotyping work by others, suggest that the level of genetic diversity in grapevine prevents the creation of a well-annotated transcriptome based on a single *V. vinifera* reference genome sequence [2, 10, 54]. We propose that a much more complete and accurate annotation can be constructed only on the basis of a cultivar-specific transcriptome assembly and a cultivar-specific genome sequence. Such genotype-specific annotations will be essential for comparative genomics of grape varieties. Only then will we be able to truly define varietal differences in grapevine on the transcriptional level.

Anchoring gene models to reference genomes and transcriptomes may be fraught with difficulties in other perennial crops as well. Although artificial selection during domestication resulted in self-compatibility in most fruit species, many of them have been derived from obligate outcrossing progenitors and, consequently, represent broad genetic diversity. Thus, variety-specific genome annotation may also become necessary to elucidate genotypic differences in such economically important fruit species as apple [55], plum [56] and sweet cherry [57]. Other highly heterozygous woody perennials for which genomics tools have been developed are cocoa [58] and poplar [59].

As our insight into the regulation of protein coding genes improves, there is mounting evidence that long non-coding RNAs (lncRNAs) play a part in regulatory processes (reviewed in [17, 60, 61]). Identification of these transcripts is paramount for understanding the role of these RNA species. We present a standardized computational pipeline for the identification of lncRNAs, which is particularly useful in non-model species. This pipeline represents a logical sequence of processes for removing known protein-coding genes and other non-coding RNAs using the most effective computational methods available to date. The pipeline predicts lncRNAs, then attempts to validate them using a pseudo-independent software, the Coding Potential Calculator. We consider this validation pseudo-independent, because both the pipeline and the Calculator incorporate BLAST results, albeit to varying levels of confidence. These transcripts are, at best, predictions, and only experimental evidence will validate their true function. This, in fact, is the major limitation of this pipeline. For example, we cannot assess the sensitivity or the specificity of the pipeline due to a lack of validated non-coding RNA sequences. This problem is inherent not only to this study, but in all current studies seeking to classify long RNA transcripts. We can, nonetheless, attempt to demonstrate that the putative lncRNAs look fundamentally different from protein coding RNA molecules. For example, our lncRNAs were overall enriched for shorter transcripts (*p* < 2.2 × 10^−16^) and lower GC content (p < 2.2 × 10^−16^). These two factors are both consistent with the previously proposed idea that lncRNAs have differential stability as compared to protein-coding transcripts [12, 45]. This result was validated by tests to determine if there was a difference in means (Welch’s t-test) and if the two metric populations were sampled from the same distributions (KS-test). At this large sample size, it was not unexpected that the results of these tests produced statistically significant differences. Nonetheless, the effect size indicated that we may be looking at true functional differences.

This pipeline is optimal for species that lack well-established reference genomes. Moreover, the pipeline works well for species that suffer from the problems presented previously, namely high heterozygosity that interferes with genome-guided methodologies. While this pipeline is well suited for such species, there is no inherent limitation of its use in other species. It is constructed in a way that it maximizes the retention of transcripts that apparently do not code for proteins regardless of the initial data. As our understanding of lncRNA biology deepens, we will return to this and other pipelines to test their efficacy in calling functionally validated lncRNAs.

## Conclusions

These and previous results of grapevine transcriptome assembly projects suggest that RNA-seq and predictive method-based genome annotation will be greatly improved by the availability of cultivar-specific genome sequences and corresponding cultivar-specific transcriptomes. This is especially necessary for the development of gene models and inter-cultivar analyses of variations. The data presented here strengthen the hypothesis that lncRNAs have thermodynamic properties that differ from those of protein-coding RNAs. The analysis of both coding and non-coding RNAs will be instrumental in uncovering inter-cultivar variation in wild and cultivated grapevine species.

## Methods

### Plant material and tissue extraction

The two accessions of *Vitis vinifera* cv. ‘Riesling’ used in this work were 588,673, a clone maintained at the USDA-ARS cold hardy grape germplasm repository in Geneva, NY and Ventosa, a commercially grown Johannisburg clone collected from Ventosa Vineyards, also in Geneva, NY. In total, tissues of seven different organs were collected from these vines representing young leaf, tendril, rachis, flower, berry (unripe and post-veraison), dormant bud, and root tissue. All tissues were collected between 10 am and 12 pm during sunny, dry conditions. In addition to field collected leaf tissue, young leaf tissue from an ongoing temperature stress experiment was collected from cuttings grown in a growth chamber (25 °C) as well as cuttings exposed to chilling temperatures (4 °C, 48 h) and freezing temperatures (−3 °C, 30 min). mRNA was isolated from each tissue-type separately using a commercially available extraction kit (Sigma Spectrum RNA kit). Following mRNA isolation and quantification, RNA pools were constructed for each genotype, equilibrated and barcoded prior to sequencing. Tissue was not available from all organs for both accessions. Thus, RNA pools for 588,673 included dormant bud, leaf, tendril, flower, rachis, unripe berry, and post-veriason berry. The Ventosa Vineyard sample included tendril, flower, dormant bud and root tissues as well as field-collected leaf tissue and chilling/freezing exposed leaf tissue. Barcoded RNA libraries were then sequenced as 150 bp, paired-end reads on a HiSeq2000 at Cornell University.

### Genome annotation

Raw RNA-seq reads from each sequenced library were assorted into bins corresponding to a directional, trial specific barcode using the FASTX tool fastx_barcode_splitter. Quality filtering was accomplished with fastq_quality_filter with the following parameters: -Q33 –q 25 –p 25. Barcodes were removed using fastx_trimmer with the following parameters: -Q33 -f 7. Further trimming of the adapter sequences.

(rcprAC = “AGATCGGAAGAGCGTCGTGTAGGGAAAGAGTGTAGATCTCGGTGGTCGCCGTATCATT”, rcprBC = “AGATCGGAAGAGCGGTTCAGCAGGAATGCCGAGACCGATCTCGTATGCCGTCTTCTGCTTG”) was performed with cutadapt [62] with the following parameters: –minimum-len 25 –O 3. The script for this processing is available in the listed GitHub repository. Reads from different sequencing lanes were concatenated into one FASTQ file representing all libraries sequenced from the left terminus and all libraries sequenced from the right terminus. Corresponding reads were paired with the tool pairfq_lite [19] using default parameters. Transcripts were de novo assembled with the program Trinity using the following parameters: --seqType fq --max_memory 22G --SS_lib_type FR --CPU 1. Flags for --left and --right were given both paired and unpaired reads from pairfq_lite delimited by a comma.

The unmasked *V. vinifera* PN40024 12Xv2 reference genome, all Trinity-assembled transcripts, and the Uniprot-Swiss-Prot database were passed as FASTA files to Maker for the first round of annotation. All settings were kept at default, with the exception of the “hidden setting” est_forward = 1. FASTA and gff3 files were merged across the entire genome using the *fasta_merge* and *gff3_merge* tools in the Maker suite to generate a preliminary transcriptome.

Using the preliminary Maker annotation, gene prediction models were trained for the final annotation of ‘Riesling’ data. Using all Maker-generated gene-models, we trained SNAP [27] to generate a gene prediction model following the methods described in the Maker Wiki [63]. The SNAP model was trained twice, iteratively. The final annotation was performed by passing the SNAP-trained Riesling.hmm (see Additional file 1), the Arabidopsis-derived Augustus model [28], and all Trinity-derived RNA-seq evidence. Options for SNAP and Augustus were defined in the Maker control file as instructed in the Wiki.

### Functional annotation of the gene models

Gene-model protein domains were identified against the Pfam31.0 [29] reference domain database using the program hmmscan (output declared as tab-delimited, −E 1e-05) of the HMMER v3.1b software suite [30]. Pfam to GO anchors were downloaded from the Gene Ontology project’s website (http://geneontology.org/external2go/pfam2go). the. Gene model searches against the Uniprot-Uniref90 and the Uniprot-Swiss-Prot protein databases were done using the blastp algorithm implemented by the BLAST suite v2.29. Results were filtered for the top hit of each query based on bit score, E-value, and percent alignment. BUSCO analyses were executed against the empryophyta_odb9 reference single-copy ortholog data set.

### Gene duplication

Duplicate genes were identified using an all-by-all self-blastp using the program MCScanX [35] with the following parameters: $path/to/MCScanX. /self_blast -e $i, where *i* represented the threshold E-value. Gene were classified into categories using the duplicate_gene_classifier algorithm implemented by the MCScanX tool. Tandem duplications were further identified from the .tandem output file. From these, TAGs were identified using the second gene in a tandem pair as an anchor for the identification of 3-gene arrays.

### lncRNA identification pipeline

The output from Trinity was filtered for redundant transcripts using the cd-hit-est. algorithm of CD-HIT [36] with the following parameters: -i Trinity.fasta -n 5 -o clust_Trinity.fasta -c 0.90 -m 8000 -T 6. Filtering by expression was executed with RSEM [37] implemented by the Trinity-provided script align_and_estimate_abundance.pl with the following flags: --seqType fq --transcripts clust_Trinity.fasta --SS_lib_type FR --est_method RSEM --ali_method bowtie --trinity_mode --prep_reference.

### Comparison of lncRNAs and protein coding RNAs

The minimum free energy of each transcript was calculated using the rnafold algorithm implemented by the ViennaRNA-2.2.5 software package [46] using the following options: -p –d2 --noLP. Results are output to a tab delimited file in which the name of the sequence, the minimum free energy (MFE), Centroid free energy, and ensemble diversity are reported. The minimum free energies of the transcripts were then compared to the minimum free energy of a randomly selected set of putative protein coding genes as annotated by Maker. Random genes were selected to be reproducible in R using set.seed (1992), and the genes were selected to coincide with occurrence patterns of lncRNAs (146 genes from chr1, 139 genes from chr2, etc.). All statistical analyses were completed in R v3.2.3 using the standard t.test () and ks-test () functions. Effect size was calculated using the formula$d=2t/\sqrt{df}$.

## Additional files

Additional file 1: Riesling-trained Hidden Markov Model for Gene Prediction. This file is a statistical model that can be used with Maker. (HMM 45 kb)

Additional file 2: Pfam to GO Anchors in the Maker Annotation. This table details the genes identified by Maker that contained Pfam domains that could be anchored to Gene Ontology terms. (CSV 4992 kb)

Additional file 3: Predicted Functional Annotation of the Maker Annotation. This table details the best hits of the Maker-derived genes to the Uniprot-swissprot and the Uniprot-Uniref90 reference protein databases. Additional data is shown for the best match gene in the reference PN40024 annotation and the type of predicted duplication classification. (CSV 1661 kb)

## Abbreviations

BUSCO: Benchmarking universal single copy orthologs
GO: Gene ontology
lncRNA: Long non-coding RNA
nt: nucleotides
RBH: Reciprocal best hit
SOYBN: Soy bean
VITVI: *Vitis vinifera*
